# Resynchronising care: addressing sex differences in cardiac resynchronisation therapy

**DOI:** 10.1016/j.eclinm.2026.103894

**Published:** 2026-04-13

**Authors:** Laura van Pelt, Jonas Erzeel, Dominic A. Theuns, Wilfried Mullens, Rudolf A. de Boer, Pieter Martens, Chahinda Ghossein-Doha

**Affiliations:** a Department of Cardiology, Erasmus MC, Cardiovascular Institute, Thorax Center, Rotterdam, the Netherlands; b Department of Cardiology, Ziekenhuis Oost-Limburg A.V., Genk, Belgium; c Hasselt University, Diepenbeek/Hasselt, Belgium; d Jessa Hospital, Department of Cardiology, Hartcentrum, Hasselt, Belgium

**Keywords:** Cardiac resynchronisation therapy, CRT, Heart failure, Sex differences, Women's health, Equity

## Abstract

Despite compelling evidence for sex-based differences in cardiovascular disease, current guidelines for cardiac resynchronisation therapy (CRT) remain predominantly informed by male-centric data. Heart failure with reduced ejection fraction (HFrEF) is less prevalent in female individuals. As a result, female patients were less frequently enrolled in early landmark CRT trials (comprising only ∼20% of participants), thus limiting the statistical power needed to detect meaningful female-specific outcomes. Emerging evidence suggests that female patients derive greater benefit from CRT at shorter QRS durations (130–149 ms) compared to male patients (≥150 ms), particularly those with left bundle branch block and non-ischaemic cardiomyopathy. Structural, electrical, and device programming differences may contribute to enhanced reverse remodelling and better clinical outcomes in females. However, these findings are seldomly reflected in guideline recommendations and rarely impact clinical decision-making, leading to underuse of CRT in eligible female patients. This Viewpoint calls for the integration of sex-specific analyses in CRT research, sex-specific recommendations in guidelines, and enhanced clinician awareness of sex-based differences.

## Introduction

Mounting evidence demonstrates sex-based differences in the clinical presentation, pathophysiology, therapeutic response, and clinical outcomes of cardiovascular disease (CVD).^1^ Cardiac resynchronisation therapy (CRT) is an established device-based treatment for selected patients with heart failure with reduced ejection fraction (HFrEF) and electrical dyssynchrony, typically manifested as a prolonged QRS duration. It is especially effective in patients with left bundle branch block (LBBB), where delayed left ventricular (LV) activation creates mechanical dyssynchrony. By coordinated biventricular pacing, CRT aims to restore synchronous ventricular contraction, thereby improving cardiac efficiency, reducing symptoms, and decreasing heart failure (HF)–related morbidity and mortality.^2^ Although highly effective, CRT remains underutilised with as many as two-thirds of eligible patients not referred, with even lower referral rates among women.^3^ Moreover, sex differences in response to CRT are increasingly documented and suggest that women may derive significantly more benefit from CRT, even at shorter QRS durations than men.^4^^,^^5^ However, current clinical practice guidelines for CRT remain predominantly informed by evidence derived from studies enrolling largely male populations. These guidelines recommend CRT as a I-A-recommendation for patients with HF in sinus rhythm, with an ejection fraction ≤35%, and LBBB with a QRS duration of ≥150 ms, who remain symptomatic despite optimal-medical therapy. For patients with shorter QRS durations the guidelines indicate that CRT should be considered and for non-LBBB QRS morphologies CRT should or might be considered depending on the pattern of the intraventricular conduction delay.^6^^,^^7^ With few exceptions, sex-specific recommendations are not incorporated in the guidelines given the low number of women enrolled. So, there is a clear lack of robust sex-specific randomised controlled trials evaluating both the efficacy and safety of CRT. Notably, the 2023 Heart Rhythm Society (HRS)/Asia Pacific HRS/Latin American HRS cardiac pacing guideline includes a stronger, Class 1 recommendation for female patients with LBBB and a QRS duration of 120–149 ms but not for male patients.^8^

Until recently, the limited sex-specific knowledge on CRT stemmed from post-hoc subgroup analyses or observational cohort studies. Furthermore, in early landmark studies the number of women enrolled was approximately 20%.^5^^,^^9^ This low number undermines statistical power to detect clinically meaningful differences for sex as a biological variable. Notable exceptions include the Clinical Investigation on Differences in the Magnitude of CRT Response in WOmen vs MEN (BIO|WOMEN) trial, which deliberately enrolled a balanced male-to-female cohort. However, it did not account for sex differences in its inclusion and treatment criteria.^10^ Another exception is the Adaptive vs conventional cardiac resynchronisation therapy in patients with heart failure (AdaptResponse) trial which included 43% women and applied sex-dependent inclusion criteria. They included patients with LBBB according to the Strauss criteria in which male patients require a QRS duration of ≥140 ms and female patients a QRS duration of ≥130 ms.^11^ In this Viewpoint, we critically appraise current evidence on sex differences in CRT and highlight key knowledge gaps that should be addressed to inform clinical-practice guidelines and make integration of sex-specific recommendations possible, aiming at improving therapeutic equity in HF management for women.

## Search strategy and selection criteria

References for this Viewpoint were identified through searches of PubMed with the search terms “cardiac resynchronisation therapy”, “CRT”, “sex differences”, and “gender differences” from 1996 until December 2025. Articles were also identified through searches of the authors’ own files. Only papers published in English were reviewed. The final reference list was generated on the basis of originality and relevance to the broad scope of this Viewpoint.

## Sex-based differences in outcomes following CRT

### Clinical outcomes

Sex-differences in clinical outcomes following CRT have been suggested by subgroup analyses of randomised controlled trials. Women report significantly better quality-of-life outcomes, including larger improvements in patient-reported symptom relief. In the Management of Atrial fibrillation Suppression in AF-HF COmorbidity Therapy (MASCOT) trial, women had a greater reduction in symptom burden as measured by the Minnesota Living with Heart Failure Questionnaire (MLHFQ), with an average score improvement of 21·19 points in women vs 16·20 points in men at 24 months (p < 0·0001).^12^ Importantly, the MASCOT trial focuses on patients in atrial fibrillation, this limits extrapolation of sex-specific quality-of-life effects to CRT recipients in sinus rhythm.

The Multicenter Automatic Defibrillator Implantation Trial With CRT (MADIT-CRT) included patients with mildly symptomatic HFrEF and prolonged QRS duration and evaluated the effect of CRT with defibrillator therapy compared to defibrillator therapy alone. A sex-specific outcome analysis, with women comprising 24·9% of the total study population, resulted in a 69% relative risk reduction in all-cause death and/or HF events in women, compared with only 28% in men (sex-by-treatment interaction p < 0·01).^4^ In a sub-analysis for QRS duration between 130 and 149 ms, this effect maintained in women with a 70% relative risk reduction, whereas men did not experience any risk reduction.^4^ In a subgroup analysis of the Multicenter InSync Randomized Clinical Evaluation (MIRACLE) study, CRT significantly prolonged the time to first HF hospitalisation (HFH) or death compared to the control group in women (hazard ratio [HR] 0·157) while no differences were found in men. Even after adjusting for baseline characteristics such as age and aetiology.^13^ Similarly, a sub study of the Resynchronization-Defibrillation for Ambulatory Heart Failure Trial (RAFT) in moderately symptomatic HFrEF patients with QRS duration of ≥120 ms comparing the same intervention, demonstrated a significant reduction of 48% in incidence of death and HFH in women compared with men (HR 0·52, 95% Confidence interval [CI] 0·33–0·81).^14^ The AdaptResponse study, the largest and most recent CRT trial, included around 3800 patients (43% women) with strict inclusion criteria for typical LBBB and intact atrioventricular (AV) conduction.^11^ It compared adaptive LV-only pacing to conventional biventricular CRT. While no sex difference was observed for the primary endpoint (all-cause mortality or HF decompensation; HR ∼0·89 for both sexes), women had consistently lower event rates compared to men. Notably, the annual event rate was low (∼7% HFH/mortality) limiting power for subgroup analyses. The study's highly selected population (true LBBB, normal AV conduction) reduces its generalisability to broader CRT practice. However, it suggests that targeting patients with narrower QRS in LBBB may be beneficial.^11^

A meta-analysis of seven major randomised controlled CRT trials further hinted at efficacy of CRT at a shorter QRS duration.^15^ It showed that women exhibited a 46% relative reduction in HFH or death (HR 0·54, 95% Credible interval [CrI] 0·42–0·70), in contrast to 23% in men (HR 0·77, 95% CrI 0·66–0·89) (sex-by-treatment interaction p = 0·009). The reduction in HFH or death was not only more pronounced in women, it also became apparent at a shorter QRS duration (125 ms) than men (145 ms).^15^

### Reverse remodelling

Beyond clinical outcomes, reverse remodelling was consistently more pronounced in women than in men following CRT implantation. Lapidot et al. demonstrated that women were nearly four times more likely than men to exhibit acute QRS narrowing ≥20 ms after CRT implantation (odds ratio [OR] 4·45, 95% CI 1·52–13·05, p = 0·006), a marker independently associated with favourable long-term outcome.^16^ Although QRS duration reduction after CRT implantation is physiologically meaningful, it is rarely a prespecified endpoint in randomised CRT trials. When evaluated, post-hoc and secondary analyses have shown that greater QRS narrowing is associated with more favourable reverse remodelling and improved clinical outcomes, but unfortunately without sex-stratified analyses robust evidence is limited.

Echocardiographic analyses further showed greater improvements in LV ejection fraction (LVEF) and larger reductions in LV end-systolic volume (LVESV) and LV end-diastolic diameter (LVEDD) in women compared to men following CRT.^10^^,^^12^^,^^17^ In the BIO|WOMEN trial, LVEF increased by an average of 14·7% in women compared with 11·5% in men after CRT implantation (p ≤ 0·01).^10^ Similarly, in the SmartDelay determined AV optimisation: a comparison to other AV delay methods used in cardiac resynchronisation therapy (SMART-AV) trial, women experienced significantly greater LVEF improvement (absolute increase of 7·2% vs 4·9%, p < 0·001) and LVESV improvement (−13·4 mL/min^2^ vs −8·5 mL/m^2^, p < 0·001) compared to men.^17^ Importantly, these trials showed substantial sex-based differences in aetiology, with 16–36% of women vs 49–66% of men having ischaemic cardiomyopathy (ICM). However, the greater extent of reverse remodelling in women remained statistically significant after adjustment for aetiology.

This observation is further supported by an analysis of the Maastricht-Utrecht-Groningen (MUG) database including 725 CRT recipients (31% women). After six months of CRT, women exhibited a 27% reduction in LVESV compared with a 17% reduction in men (p < 0·001). Although women in this cohort less frequently had ICM than men (28·5% vs 58·8%), female sex remained independently associated with greater LVESV reduction after multivariable adjustment, including HF aetiology.^18^

**Fig. 1:**
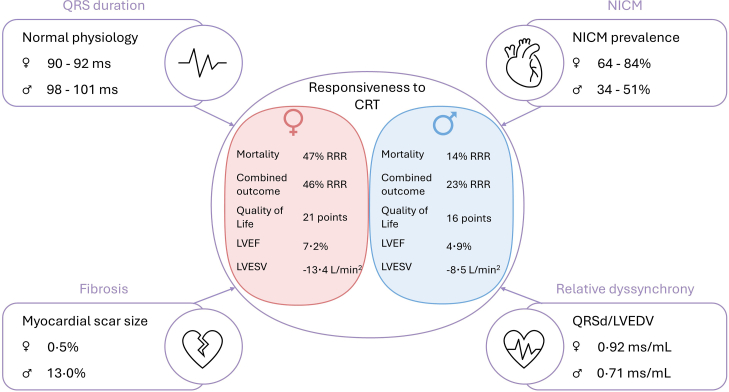
**Sex differences in responsiveness to CRT surrounded by factors that influence the responsiveness**. Combined outcome consists of mortality and/or heart failure hospitalisation. CRT: cardiac resynchronization therapy; LVEDV: left ventricular end diastolic volume; LVEF: left ventricular ejection fraction; LVESV: left ventricular end systolic volume; NICM: non-ischaemic cardiomyopathy; QRSd: QRS duration; RRR: relative risk reduction.

Together, these data indicate that while differences in underlying aetiology contribute to sex-specific CRT responses, female sex itself remains independently associated with more pronounced reverse remodelling. Fig. 1 shows an overview of the sex differences in responsiveness to CRT.

## Physiological basis for sex-based differences in QRS duration and implications for CRT thresholds

Physiological sex differences play a fundamental role in explaining the shorter QRS duration observed in women. On average, women have a QRS duration that is 4–10 ms shorter than that of men.^19^ This disparity is multifactorial in origin and may include size, differences in relative dyssynchrony, aetiology and fibrosis burden. An overview of sex differences in factors that influence the responsiveness to CRT can be found in Fig. 1.

### Size

First, women have a smaller body surface area (BSA), which correlates with a smaller cardiac size, including reduced LV mass and shorter ventricular conduction pathways.^20^ Second, women exhibit a lower total LV volume and myocardial fibre length, which contributes to more rapid completion of ventricular depolarisation.^20^ Additionally, some studies suggest that intrinsic electrophysiological differences, such as a higher resting membrane potential and potentially faster myocardial conduction velocities in women, may also contribute to shorter QRS durations independent of cardiac size.^21^

A meta-analysis of two large CRT trials indicated that smaller patients receive a greater benefit from CRT. This interaction was observed between the effects of CRT and BSA (HR 0·50, 95% CI 0·38–0·66, p < 0·0001) but not with body mass index. Although this analysis did not find a sex difference, they do note that it cannot be completely ruled out.^22^

Another meta-analysis showed that while indexing QRS duration by body size attenuated the differences in CRT response between men and women, it did not abolish them.^15^ Potential explanations for the remaining sex differences include a difference in functional block location or differences in scarring, septal wall thickness or conduction via middle septal fascicle.^15^

### Relative dyssynchrony

Relative dyssynchrony, quantified as QRS duration normalised by LV end-diastolic volume (LVEDV) or LV mass, has emerged as a more precise predictor of CRT responsiveness than QRS duration alone. This ratio captures the concept that conduction delay over a smaller myocardial volume implies greater inefficiency, and thus higher potential for therapeutic resynchronisation. A mechanistic explanation for the sex-specific benefits lies in the degree and timing of mechanical dyssynchrony. A greater relative electrical dyssynchrony appears to lead to a greater mechanical dyssynchrony. De Pooter et al. demonstrated that women with LBBB not only present with greater dyssynchrony but also manifest it at shorter QRS durations than men.^23^ Since CRT targets mechanical dyssynchrony, the earlier manifestation of this substrate in women may explain their greater responsiveness at shorter electrical delays. The women in the MOre REsponse on Cardiac Resynchronization Therapy With MultiPoint Pacing (MORE-CRT MPP) trial did have a significantly smaller LVEDV (176 ± 60 mL) than men (228 ± 77 mL, p < 0·0001) yet displayed a higher CRT response rate (70·1% vs 56·8%, p < 0·0001). Importantly, women exhibited significantly greater relative electrical dyssynchrony, as measured by the QRSd/LVEDV ratio (0·92 [0·73–1·13] in women vs 0·71 [0·59–0·89] in men, p < 0·001).^24^

Similarly, Salden et al. proposed indexing electrical dyssynchrony to LV size to enhance the discriminatory power of QRS metrics, advocating a personalised threshold approach that accounts for interindividual anatomical variability.^18^

These data underscore that sex and/or cardiac size differences in raw QRS duration may obscure underlying physiological disparities in conduction efficiency and myocardial geometry. For women with inherently shorter baseline QRS durations, reaching the 150 ms threshold represents a proportionally greater prolongation and may therefore indicate a more advanced stage of dyssynchrony by the time CRT is considered. Consequently, applying sex-neutral criteria risks delaying intervention in women who might already be experiencing substantial mechanical dyssynchrony at shorter QRS durations. This may partly explain why several studies and modelling analyses, such as those by Lee et al., show that CRT potentially becomes beneficial for female patients at QRS durations 9–13 ms shorter than the guideline-recommended threshold.^25^

### Aetiology and fibrosis

Women with HF more frequently have non-ischaemic cardiomyopathy (NICM), whereas men more often present with ICM, a pattern consistently reflected across large cohorts and clinical trials.^26^ Moreover, patients with NICM appear to have a better CRT response compared to ICM, possibly due to less myocardial fibrosis leading to improved ventricular conduction.^27^ Studies have shown that women with NICM have significantly smaller myocardial scar sizes than men (0·5% vs 13·0%).^28^ In the case of ICM, women appear to have similar fibrosis compared to men.^28^ In the NICM subgroup of the MORE-CRT MPP study, relative dyssynchrony was independently predictive of CRT response (p = 0·0039) while sex was not.^24^ A large meta-analysis reported that CRT in NICM was associated with a larger improvement in LVEF as well as LVEDD and LV end-systolic diameter compared to ICM. They suggest that this difference could be the result of patients with NICM having more viable cardiac tissue that can act as a substrate for CRT. Despite the larger improvements in patients with NICM, the time to HFH or death after CRT implantation increased similarly for both aetiologies. The proposed explanation is a different cause of the CRT benefit, with NICM benefiting due to reverse remodelling and ICM through delaying adverse remodelling. While women were only 25% of the included patients, the meta-analysis did also note that for patients with NICM the reduction of HFH or death was greater for women than men.^29^

## Sex-based response in device optimisation

Beyond anatomical and structural factors, compelling evidence indicates that sex-based differences extend to the electrophysiological response and device programming in CRT. Importantly, female patients are more likely to receive a higher percentage of effective biventricular pacing likely reflecting a more favourable electrical substrate, including a higher prevalence of true LBBB, lower atrial fibrillation burden, smaller ventricular size, and less myocardial scar.^12^^,^^17^ This increased biventricular capture has been directly associated with superior reverse remodelling, particularly greater reductions in LVESV. Moreover, AV delay optimisation, an often-underutilised aspect of CRT programming, has shown sex-specific benefit. AV optimisation refers to adjusting the timing between atrial and ventricular contraction in patients receiving CRT, in order to maximise ventricular filling and improve cardiac output. Proper AV timing helps ensure that the ventricles contract at the most effective moment after atrial contraction, thereby enhancing the haemodynamic benefit of CRT.

In women, AV optimisation led to significantly greater improvements in LVESV while this enhancement was not observed in male patients, and interaction analyses confirmed a statistically significant sex-by-optimisation effect, even after adjustment for baseline covariates (p = 0·025).^17^

These findings suggest that women not only benefit from CRT at different electrical thresholds but also respond more favourably to tailored device programming. This underscores the importance of incorporating sex-specific considerations into both CRT implantation criteria and post-implantation management strategies, including individualised AV delay settings and pacing strategies. Failure to account for these differences risks suboptimal outcomes in a population that demonstrably stands to gain substantial benefit from optimisation.

## Clinical implications and future directions

Sex-based differences in CRT response are robust and clinically meaningful yet remain obscured by reliance on male-dominant trial data and uniform QRS thresholds. For women who typically have shorter baseline QRS durations, waiting until the 150 ms threshold may already represent advanced dyssynchrony and a missed opportunity for earlier intervention. Although much of the evidence is derived from post hoc analyses and is therefore limited in statistical power, converging data from multiple studies suggest that women benefit from CRT at QRS durations approximately 9–13 ms shorter than current guideline thresholds. Another factor to consider is ethnicity, as this can also influence electrical parameters such as QRS duration, for instance patients of Asian descent are overall smaller in body size and have shorter QRS durations.^30^ A recalibration of selection criteria, informed by sex, body size, and ethnicity, is needed to avoid systematic undertreatment of women and to ensure that CRT is delivered at the point of maximal benefit rather than at the stage of irreversible disease.

**Fig. 2:**
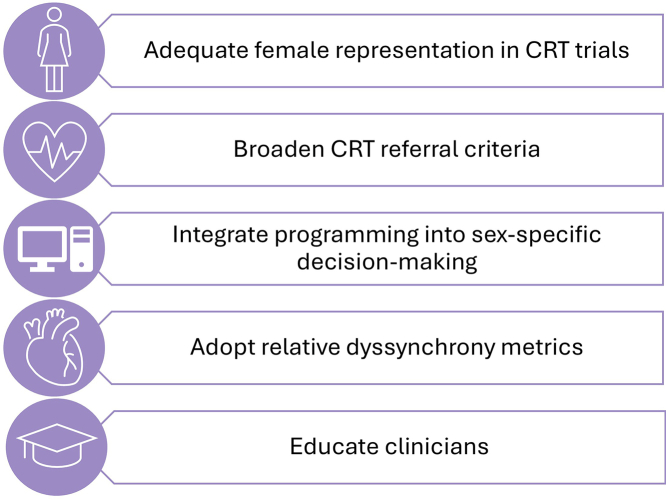
**Future directions for the improvement of therapeutic equity in heart failure management for women**. CRT: cardiac resynchronization therapy.

To translate this evidence into clinical benefit, several actions are essential (Fig. 2).

1. Ensure adequate female representation in CRT studies. Future trials must be powered for sex-specific analyses, with predefined stratification and enrolment targets. Inclusion of proportional female cohorts and adjustments for key variables such as aetiology and LV size are critical and should be considered and reported.
2. Broaden CRT referral criteria for women. Current QRS duration thresholds should be re-evaluated. Women with LBBB and QRS durations between 130 and 149 ms have shown comparable, if not superior, outcomes to men with QRS ≥150 ms. Guidelines must incorporate sex-specific thresholds to avoid under-referral.
3. Integrate CRT programming into sex-specific decision-making. Despite women benefitting more from AV optimisation and receiving more effective biventricular pacing, CRT programming is still often underutilised. Individualised device programming should be a standard consideration, particularly in female patients.
4. Adopt relative dyssynchrony metrics. Using QRS duration indexed to LV size (e.g., QRSd/LVEDV) may offer a more physiologically relevant selection criterion than absolute QRS duration alone and should be validated in prospective studies.
5. Educate clinicians. Lack of awareness remains a major barrier. Targeted education initiatives and integration of sex-specific evidence into clinical training and practice tools are essential to achieving equitable and evidence-based CRT delivery.

## Conclusions

In conclusion, addressing sex disparities in CRT is not only a matter of equity but a crucial step toward precision medicine in cardiac electrophysiology. Doing so will ensure that all patients, regardless of sex, receive the full therapeutic benefit of device-based HF therapies. It is time to resynchronise our criteria to achieve equity in CRT.

## Contributors

LP conceptualised the manuscript, performed literature search, wrote the initial draft of the manuscript, and continued editing further drafts. JE conceptualised the manuscript, performed literature search and continued reviewing and editing further drafts. DT, WM, and RB contributed to the reviewing of subsequent drafts PM and CGD contributed to the conceptualisation of the manuscript and reviewing of subsequent drafts.

## Declaration of interests

The institution of Prof. dr. De Boer, CGD, DT, and LP has received research grants and/or fees from Alnylam, AstraZeneca, Abbott, Bristol-Myers Squibb, NovoNordisk, and Roche; Prof. dr. de Boer has had speaker engagements with and/or received fees from and/or served on an advisory board for Abbott, AstraZeneca, Bristol-Myers Squibb, NovoNordisk, Roche, and Zoll. Prof. dr. de Boer reports on a patent on the use of circulating bone morphogenic protein 10 in the assessment of congestion and pulmonary hypertension (WO2025061752). Prof. dr. de Boer is a board member of the ESC Councill on Cardiovascular Genomics, a past president of the Dutch Cardiac Society, and an associate editor of the European Heart Journal. Prof. dr. Martens has had speaker engagements with and/or received fees from Bristol-Myers Squibb and NovoNordisk. All other authors declare no competing interests.
